# Gastrointestinal Fluid Volumes in Pediatrics: A Retrospective MRI Study

**DOI:** 10.3390/pharmaceutics14091935

**Published:** 2022-09-13

**Authors:** Matthias Van der Veken, Michael Aertsen, Joachim Brouwers, Cordula Stillhart, Neil Parrott, Patrick Augustijns

**Affiliations:** 1 Drug Delivery and Disposition, KU Leuven, Gasthuisberg O&N II, Herestraat 49—Box 921, 3000 Leuven, Belgium; 2 Department of Imaging and Pathology, Clinical Department of Radiology, University Hospitals KU Leuven, 3000 Leuven, Belgium; 3 Formulation & Process Sciences, F. Hoffmann-La Roche Ltd., 4070 Basel, Switzerland; 4 Pharmaceutical Sciences, Roche Pharma Research and Early Development, Roche Innovation Centre Basel, 4070 Basel, Switzerland

**Keywords:** MRI, biorelevant dissolution, PBPK, gastric fluid, intestinal fluid, pediatrics

## Abstract

The volume and distribution of fluids available in the gastrointestinal (GI) tract may substantially affect oral drug absorption. Magnetic resonance imaging (MRI) has been used in the past to quantify these fluid volumes in adults and its use is now being extended to the pediatric population. The present research pursued a retrospective, explorative analysis of existing clinical MRI data generated for pediatric patients. Images of 140 children from all pediatric subpopulations were analyzed for their resting GI fluid volumes in fasting conditions. In general, an increase in fluid volume as a function of age was observed for the stomach, duodenum, jejunum, and small intestine (SI) as a whole. No specific pattern was observed for the ileum and colon. Body mass index (BMI), body weight, body height, and SI length were evaluated as easy-to-measure clinical estimators of the gastric and SI fluid volumes. Although weight and height were identified as the best estimators, none performed ideally based on the coefficient of determination (R^2^). Data generated in this study can be used as physiologically relevant input for biorelevant in vitro tests and in silico models tailored to the pediatric population, thereby contributing to the efficient development of successful oral drug products for children.

## 1. Introduction

Although one of the oldest forms of drug delivery, oral administration continues to be the preferred route for drug therapy. In the past few decades, extensive research has been performed to map and characterize the complex processes that determine drug absorption, including drug release, dissolution/precipitation, solubilization, and permeation [1,2]. Such research has advanced the development of more biorelevant in vitro tools to simulate, for instance, drug dissolution and solubility [3]. Additionally, the research contributed to the development of physiologically based pharmacokinetic (PBPK) absorption models where these processes are described mechanistically and mathematically, enabling computational simulation of drug absorption. Both biorelevant in vitro dissolution testing and PBPK absorption modeling play a critical role in the efficient development of oral drug products [3,4,5]. The ability of such tools to predict drug absorption in humans, however, depends on the input of physiological reference data. For example, to give a suitable prediction of the absorption process in PBPK modeling, a thorough description of the gastrointestinal (GI) tract is required [6]. Biorelevant dissolution setups on the other hand require input on the use of physiologically relevant fluids and fluid volumes [2].

Even though several absorption-related physiological factors have been investigated, research has mainly focused on the adult population, largely neglecting specific populations such as children [1]. This lack of characterization is apparent for both in vitro and in silico setups used in pediatric drug development, where scaling is often performed allometrically instead of based on physiological reference information. For variables where no data are available, no scaling might be applied [7]. These approaches limit the biorelevance and predictive value of absorption simulation and may therefore result in the development of oral drug products for children that have suboptimal drug performance in vivo.

For the pediatric population, regulatory authorities acknowledged this gap and reacted with Regulation No 1901/2006, the Pediatric Research Equity Act (PREA), and the Best Pharmaceuticals for Children Act (BPCA) [8,9,10]. As of 2002, these regulations and acts set out the requirement for a more extensive investigation of the pediatric population and the in vivo performance of (newly developed) drugs. Since then, multiple differences in physiology and biopharmaceutics between children and adults have been identified [1,11]. With respect to the GI environment in children, exploratory studies have already shown that it differs from the adult environment considering pH, bile salt content, osmolality, and fluid volume [1,12,13,14]. However, more extensive research is needed to expand and validate the limited physiological reference data currently available and to integrate these physiological data into reliable simulation tools and models.

A physiological variable with a potentially substantial impact on drug absorption is the volume of GI fluids. It is quite evident that a larger volume available for dissolution allows more drug to dissolve and become available for absorption. For bioavailability and bioequivalence studies in adults, regulatory and drug development guidelines advise administration of drugs with 150–240 mL of water [15,16,17]. In practice, however, drugs are often administered with smaller volumes of water or no fluid at all [18]. This makes knowledge of resting fluid volumes in the GI tract even more crucial to understand drug behavior and absorption. Additionally, it is important to understand the distribution of fluid over the GI tract, since sufficient fluid should be available at the absorption site of the drug. In this respect, magnetic resonance imaging (MRI) has already proven its value to localize and quantify fluid volumes in the stomach and intestine of adults [17,19,20,21,22], providing valuable input data for biorelevant in vitro dissolution tests and PBPK modeling [17,23].

While MRI has been extensively used in the past to determine GI fluid volumes in adults, its use is now being extended to the pediatric population, with two recent small-scale studies analyzing the stomach and small intestine (SI) (32 fasted children, aged 0–16 years) and the colon (28 fasted children, aged 0–15 years) [13,14]. The limited data currently available show both a high variability in fluid volumes among children and important differences compared to adults. To further close the knowledge gap regarding pediatric GI fluid volumes, the present research pursued a retrospective, explorative analysis of existing clinical MRI data generated for pediatric patients at the University Hospitals Leuven (Belgium). This study aimed to extend the available data on resting GI fluid volumes in fasted children, which can be used as input for biorelevant in vitro tests and PBPK models tailored to the pediatric population. As such, fluid volumes were quantified along the entire GI tract considering all different segments. Taking into account the heterogeneity of the pediatric population, data were analyzed per pediatric subpopulation as defined by the International Conference on Harmonization (ICH) [24].

## 2. Materials and Methods

### 2.1. Ethics

This study was a retrospective observational study held at the University Hospitals Leuven and approved by the Ethics Committee Research UZ/KU Leuven (S64993—Quantification of gastrointestinal fluid volumes in the pediatric population using MRI—approved 25 January 2021).

### 2.2. Patient Selection

All pediatric abdominal MRI images obtained in children aged 0–16 years at the University Hospitals Leuven between 1 January 2010 and 1 August 2020 were evaluated for inclusion. All data prior to this date were excluded to reduce the effect of variability in the MRI acquisition protocol. Children older than 16 years are handled as adults when imaging at the University Hospitals Leuven and were therefore not considered. Only images obtained in fasted state conditions were included, excluding all postmortem imaging and imaging that requires a fluid-fed state. As described by Papadatou-Soulou et al. [14], fluid-fed state imaging is a type of imaging where children are given large volumes of osmotically active fluid as a contrast agent. Due to the osmotic activity and volume of this fluid, children in the fluid-fed state were not considered to be in a physiological reference state.

Although the hospital MRI protocol requires a fasting period of 6 h, it could not be excluded that some patients were not fully fasted. Especially younger children tend to get irritated when being fasted and are often allowed some food [25]. Additionally, some scans may have been taken in an emergency setting with a shorter or absent fasting period. For these reasons, the fasted state of the patients was verified based on the characteristics of the stomach. In line with Koziolek et al. [26], the following visual criteria for a fed state were used: expansion of the stomach wall (an expanded stomach and consequently thinner gastric wall indicate a fed state), characteristics of the gastric contents based on the signal intensity on T2-weighted images (fasted state: homogeneous and hyperintense signal representing free fluid; fed state: heterogeneous rather hyper- to iso-intense signal representing a mixture of solid food and fluid).

**Figure 1:**
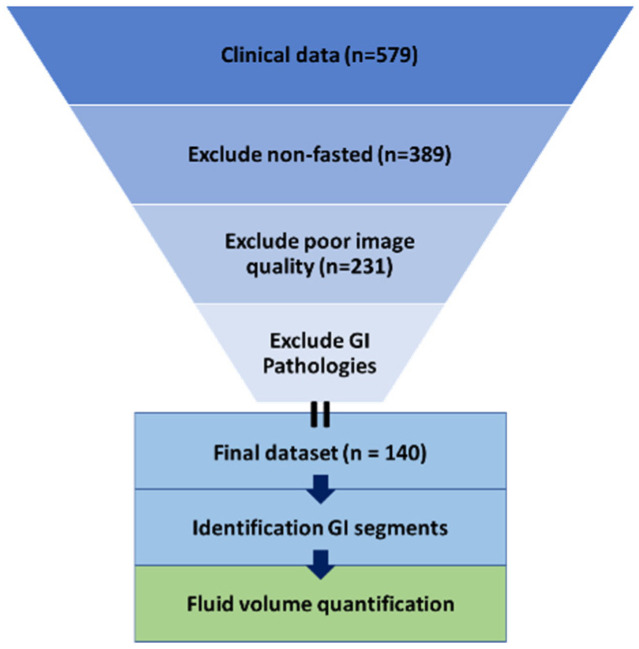
Visualization of the methodology used.

Next, images with insufficient quality were excluded. This selection involved exclusion based on image contrast, image resolution, motion degradation, and incomplete visibility of the GI tract. Lastly, patients were excluded based on the presence of pathologies that may affect the fluid in the GI tract (cholecystectomy, pancreatitis, large tumors or cysts putting pressure on the GI tract, children with a stoma after previous bowel surgery, Crohn’s disease, ulcerative colitis, extensive splenomegaly or hepatomegaly). The excluded number of patients per criterium is summarized in Figure 1 and Table 1. The final dataset contained images from 140 patients with no pathologies expected to affect the GI fluid volumes.

### 2.3. MRI Fluid Integration

All MRI images included in the analysis were obtained using a clinical magnet of 1.5 Tesla (T) (Siemens Aera, Erlangen, Germany or Philips Medical Systems Achieva dStream, Koninklijke Philips NV, Best, the Netherlands) or 3T (Philips Medical Systems Ingenia, Koninklijke Philips NV, Best, the Netherlands). The images were acquired following the routine clinical protocol in the axial and coronal planes using either T2-weighted ultrafast spin echo sequences (half-Fourier acquisition single-shot turbo spin echo (HASTE) or T2-weighted single-shot turbo spin echo sequences (SS-TSE)) or T2-weighted steady-state balanced gradient echo sequences (True FISP or Balanced Fast field echo). Over the 10-year period considered for data selection, only minimal adjustments were made to the mentioned sequences to improve image quality after system upgrades; therefore, all generated data were considered comparable.

Images were analyzed to calculate fluid volumes in the GI tract using Horos^TM^ (Version 3.3.6, Horosproject.org sponsored by Nimble Co LLC d/b/a Purview, Annapolis, MD USA), a free and open-source code software (FOSS) medical image viewer. Integration of free fluid was performed in accordance with the protocol published by de Waal et al. [22]. In brief, free fluid has a high signal intensity on T2-weighted images. For every scan, a threshold for free fluid was defined as 30–40% of the signal intensity of the brightest voxels located in cerebrospinal fluid in the spinal canal and bile in the gallbladder, as these can be considered as definite free fluid. During the integration of the fluid pockets in the GI tract, the threshold was adapted manually between this 30–40% mark to obtain a visual contour that fully covered the individual fluid pockets. Manual corrections were performed to exclude the fluid outside the GI tract (e.g., gallbladder, urinary tract, spinal canal, interstitial fluid). The integration did not include the heterogeneous hyper- to iso-intense fluid that is present in the distal small and large intestine as digested food [19,22].

For all included patients, fluid volumes were quantified over the whole GI tract. Fluid pockets in the stomach, duodenum, jejunum, ileum, and colon were segmented separately. Using the integrated area of the fluid pockets and the slice thickness with the interslice gap, the respective volumes in the GI segment were calculated.

### 2.4. MRI Gastrointestinal Segment Identification

**Figure 2:**
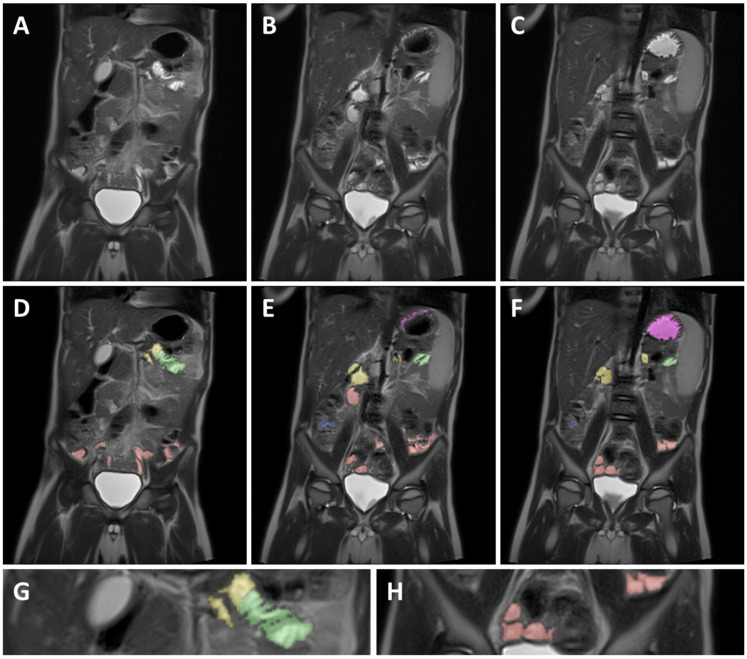
Example of MRI identification of fluid pockets in different GI segments on 3 coronal T2 weighted spin echo images of a 5-year-old child. (**A**–**C**) are unmarked images; (**D**–**F**) are the corresponding marked images. Purple marked areas are located in the stomach, yellow in the duodenum, green in the jejunum, red in the ileum, and blue in the colon. (**G**) is a detailed view of the jejunum (green), showing the circular folds as the typical striped pattern in the fluid pocket. (**H**) is a detailed view of the ileum marked in red, showing the smooth intestinal wall.

To ensure consistency, different GI segments were identified based on the following identification criteria for each segment. Identifications of the different GI segments are described from the patient’s perspective. An example can be found in Figure 2.

#### 2.4.1. Stomach

When dividing the body using an X, Y coronal plane, the stomach is located in the top left quadrant and passes over the *Y*-axis to the top right quadrant. Due to the presence of often larger and localized fluid volumes, it is relatively easy to identify the stomach and integrate the fluid volume. The pylorus was identified as the end of the stomach and served as a marker for the start of the duodenum.

#### 2.4.2. Duodenum

The duodenum starts at the duodenal bulb immediately after the pylorus and then typically follows a path down, after which the duodenum crosses the *Y*-axis of the coronal plane and continues up again until the ligament of Treitz. The ligament of Treitz, and thus the end of the duodenum, was identified as being roughly the same height as the duodenal bulb but on the left side of the spine [27].

#### 2.4.3. Jejunum

As commonly accepted, the jejunum starts at the ligament of Treitz [27]. The end of the jejunum and transition to the ileum, however, is hard to define precisely using MRI imaging [27]. Consequently, the morphology and relative position in the abdominal cavity was used to differentiate between the jejunum and ileum. Morphological differentiation was based on the *valvulae conniventes*. Due to the closely spaced circular folds and the consequently thicker intestinal wall, the jejunum can be identified by a typical striped pattern in the GI fluid [27] (Figure 2G). In addition, the jejunum is mainly located in the top left quadrant, sometimes extending into the bottom left quadrant of the abdomen, while the ileum is mainly located in the bottom right quadrant, sometimes extending into the bottom left quadrant.

#### 2.4.4. Ileum

Contrary to the jejunum, the ileum has less closely spaced circular folds [27] (Figure 2H). Consequently, the ileum can be identified by a smoother lined and thinner intestinal wall compared to the jejunum. The ileum ends with the ileocecal valve located at the start of the well-defined and easily localized colon.

#### 2.4.5. Colon

The colon is the last part of the GI tract and has a larger diameter as compared to the jejunum and ileum. Additionally, the position of the colon is more fixed [27], therefore allowing for easier identification and localization. The colon follows a well-defined path starting after the ileocecal valve in the lower right quadrant (caecum) and going up (ascending colon). Next, the colon crosses the central *Y*-axis (transverse colon), after which it comes back down (descending colon) to end as the sigmoid and rectum.

### 2.5. Potential Impact of Small Intestinal Fluid Volumes on Drug Absorption Using PBPK Modeling

**Figure A1:**
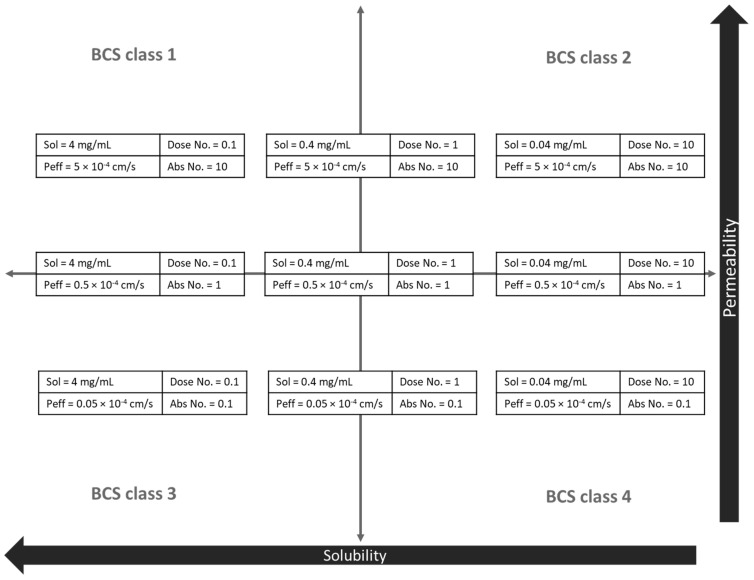
Overview of the hypothetical drug characteristics used in the PBPK sensitivity analysis (Figure 6). Sol = solubility, Peff = effective permeability.

PBPK models for hypothetical compounds were developed in Gastroplus (Version 9.8, Simulation Plus, Inc., Lancaster, CA, USA). The default values of input data for a newly created drug record in Gastroplus were taken except for the solubility at pH 7 and the effective permeability. Based on the requirements for the different biopharmaceutical classification system (BCS) classes and the Gastroplus generated dose number and absorption number, 9 hypothetical neutral drugs with a 100 mg dose were generated, representing the 4 different BCS classes complemented by 5 boundary BCS class drugs. The dose number is a non-dimensional number calculated by dividing the dose by the amount that will dissolve in the volume of co-administered fluid (typically 250 mL of water) at the lowest solubility between pH 1 and 8 [28]. A dose number of 10, 1, and 0.1 represents a low, moderately, and highly soluble drug, respectively. The absorption number is also a non-dimensional number, calculated by dividing the SI transit time by the SI absorption time, and reflects the effective permeability [28]. An absorption number of 10, 1, and 0.1 represents a high, moderate, and low permeable compound, respectively. The corresponding solubility and permeability values for the different hypothetical drugs can be found in Figure A1. All other input data are available in Table A1.

Next, a manual sensitivity analysis of the simulated fraction absorbed (Fa%) as a function of SI fluid volume was performed for each of the compounds. GI fluid volumes in Gastroplus can be set by specifying the % of the total segmental volume filled with fluid.

## 3. Results and Discussion

To extend the knowledge on GI fluid volumes in the different pediatric subpopulations, the present study opted for a retrospective analysis of lower abdomen MRI data from pediatric patients obtained for diagnostic purposes in clinical practice. Even though such a study design does not allow full control over the experimental conditions, it provides an opportunity to obtain relevant physiological data in vulnerable populations for which prospective studies are challenging due to ethical concerns.

### 3.1. Population

To ensure the relevance of the obtained data, a careful selection of available patients and images was performed. As described in the Methodology section, patients with pathologies expected to affect the GI environment and patients in a non-fasted state were excluded. After applying the exclusion criteria, MRI data from 140 patients were evaluated for GI fluid volumes. Demographical data from the included patients can be found in Table 2. Overall, the included patients had an average body mass index (BMI) of 17.30 kg/m^2^; 52.14% of them were female. The majority of the patients (58.57%) were preschool children aged 2–5 years, but the other pediatric subpopulations were also represented. Most of the patients (51.43%) had an oncology-related diagnosis or history, with the majority being patients with neuroblastomas in regression. Oncology was followed by urogenital (21.43%) and hepatic (20.71%) pathologies. Lastly, 6.43% of the patients received other diagnoses, such as lymphatic and cardiovascular issues. The pathologies of the included patients, extracted from their medical history records, were assumed not to affect the fluid volumes in the GI tract. As per the MRI protocol of the University Hospitals Leuven, MRI was performed under anesthesia for patients under the age of 6 years.

### 3.2. Gastrointestinal Fluid Volumes

**Figure 3:**
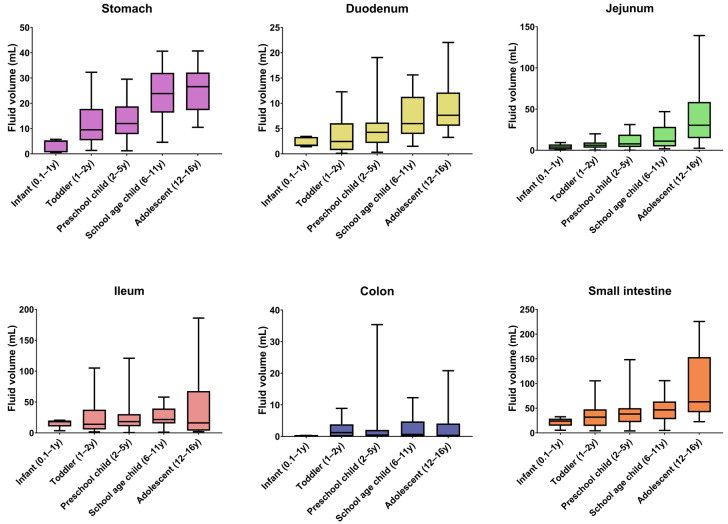
GI fluid volumes per segment and per pediatric subpopulation. Boxes indicate the 25th and 75th percentile and the median. Whiskers indicate the minimum and maximum. The number of children per subpopulation can be found in Table 2 (y = year).

The median GI fluid volumes for each pediatric subpopulation are summarized in Figure 3, together with the full and interquartile range. The median resting fluid volumes increased with age for all GI segments, except for the ileum and the colon, for which no specific pattern was observed. For the stomach, median resting fluid volumes increased from 5.00 mL for infants to 26.58 mL for adolescents. For the full SI, an increase from 23.87 mL in infants to 62.90 mL in adolescents was observed. In general, median fluid volumes increased going more distally in the SI when comparing duodenum, jejunum, and ileum. Only in the adolescent subpopulation was the median fluid volume lower in the ileum than in the jejunum. For the colon, only the infant subpopulation (0.1–1 year) seemed to have a lower resting fluid volume than the other subpopulations. Overall, the colonic fluid volumes were characterized by a broad variability. This high variability in colonic fluid volumes originated from the presence of either no (0 mL) or one larger fluid pocket (>5 mL) in some patients.

In the stomach, the resting fluid volume was present as a bright homogeneous and continuous fluid pocket, which is in contrast to the rest of the GI tract. As described by Mudie et al. [17], intestinal fluid in adults is not present as a continuous fluid but rather as individual localized pockets. This was also observed in children, both in the present study and by Papadatou-Soulou et al. [14]. It should be noted that the observed gastric and duodenal fluid pockets were mostly located toward the fundus and corpus of the stomach and the duodenal bulb, the descending and the ascending part of the duodenum. As MRI imaging is performed with the patient lying down, these intestinal parts are located lower compared to the others. When standing or sitting (which might be more relevant for drug administration), more fluid might be present in different GI segments such as the transient duodenum. To investigate this effect of gravity on the location of fluid pockets, MRI imaging of people standing up as used in, for instance, lumbar imaging [29] would be a valuable tool but until today these are only available in low magnet strength (<1 T) limiting their applications.

In line with the physiological function of the colon to reabsorb water [27], most colonic fluid pockets were located in the ascending colon and sometimes in the transverse colon. In the descending part of the colon, very few and small to no fluid pockets were observed, indicating complete reabsorption of free fluid. This fluid pocket location pattern in children was also observed by Goelen et al. [13].

### 3.3. Correlation of Gastrointestinal Fluid Volumes with Body Characteristics

The obtained data from 140 children allowed evaluation of the correlations between the GI fluid volumes and easy-to-measure body characteristics. Such correlations may provide the basis for simple estimations of the GI fluid volume by identifying a normalization factor or clinical estimator. These normalized fluid volumes can then be used for more representative and tailored dissolution studies [30]. The evaluated body characteristics of the patients, including body weight, body height, BMI, and SI length, were all evaluated for their correlation with the measured gastric and SI fluid volumes. The SI length was predicted based on body height as described by Struijs et al. [31], who performed an in situ measurement of the SI length starting from the ligament of Treitz to the ileocecal valve using a silk suture. As the length of the duodenum is relatively small compared to the rest of the SI, this predicted length was taken as representative of the whole SI.

**Figure 4:**
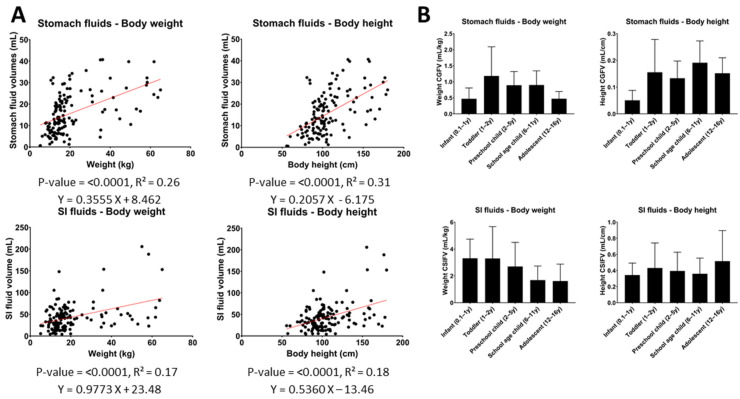
(**A**) Correlations between gastric or SI fluid volumes and body weight and height. (**B**) Weight- or height-normalized gastric/SI fluid volumes per pediatric subpopulation. (Two-tailed *p*-value of correlation, R^2^ = coefficient of determination, CGFV = corrected gastric fluid volume, CSIFV = corrected SI fluid volumes, y = year).

Even though a statistically significant linear correlation was found between all tested body characteristics and the GI fluid volumes, none of the respective coefficients of determination (R^2^, calculated as the square of the correlation coefficients) indicated a reliable prediction. The strongest correlations were found between GI fluid volumes and body height or weight, for which R^2^-values ranged between 0.17 and 0.31, which is still poor (Figure 4A). For the other tested body characteristics, BMI, in general, performed the worst with an R^2^ of 0.09 and 0.03 for stomach and SI fluid volumes, respectively. For the SI length, the R^2^ with SI fluid volumes was 0.16.

The relatively poor correlations imply that the added value of normalizing GI fluid volumes by any of these body characteristics is rather limited. This is illustrated in Figure 4B, which shows that, after normalization of the measured GI fluid volumes, an increasing or decreasing trend with age or a relatively high variability between different subpopulations still remained.

### 3.4. Literature Comparison

#### 3.4.1. Stomach

The measured gastric fluid volumes in the present study differed from the volumes measured by Papadatou-Soulou et al. [14], who reported an average gastric fluid volume of only 1.3 mL for children aged between 0 and 16 years old, with 65.6% of children having even no gastric fluid present. These low volumes strongly contrast with the present study in which resting fluid was detected in the stomach of all children, with an average volume for all combined subpopulations (age 0.1–16 years) of 15.04 mL. A comparison per subpopulation was not possible, as Papadatou-Soulou et al. reported an average fluid volume for the whole pediatric population. The discrepancy in average gastric fluid volumes might originate from differences in the applied integration technique. In the studies of both Papadatou-Soulou et al. [14] (upper GI tract) and Goelen et al. [13] (lower GI tract), a fully automated approach to set the threshold for free fluid integration was used, thereby allowing for a more computerized analysis. The threshold was set based on the average signal intensity of the cerebrospinal fluid. This average, however, is sensitive to outliers such as very bright or dark voxels, which might originate from movement artifacts. A possibly higher threshold might have reduced the sensitivity of fluid integration. In the current study, the threshold was manually optimized per image set to reduce the impact of such artifacts. Additionally, while the more automated approach allows for faster image handling, it could also miss some smaller fluid pockets.

**Figure 5:**
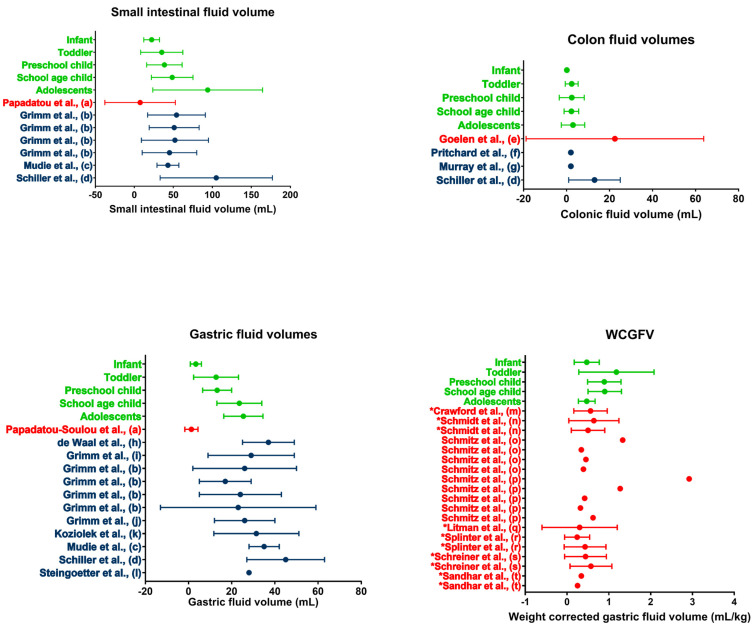
Literature comparison for SI, colon, gastric, and WCGV. Dots indicate average volumes; error bars indicate standard deviations. Data obtained in the present study (green) are compared to data from other studies on children (red) or adults (blue). WCGV measured from fluid aspirations are indicated by a * in front of the reference; other WCGVs are measured using MRI. (a [14], b [20], c [17], d [31], e [13], f [32], g [33], h [22], i [21], j [35], k [26], l [36], m [37], n [38], o [39], p [40], q [41], r [42], s [43], t [44]).

While data regarding fasted gastric fluid volumes in the pediatric population are limited, a larger dataset is available for the adult population. A comparison with available average gastric fluid volumes in the literature shows that the gastric fluid volumes measured for adolescents (25.39 ± 9.08 mL) and school-age children (23.52 ± 10.40 mL) are in line with the results available for adults (Figure 5).

#### 3.4.2. Small Intestine

As this is, to the best of our knowledge, the first time that jejunal and ileal fluid volumes are determined separately using MRI, comparison with literature data is difficult. One exception to the integration of fluid pockets in individual SI segments is de Waal et al. [22], who measured an average duodenal volume of 12.62 ± 7.76 mL in healthy adults. These duodenal data are in line with the average volumes measured for the adolescent population in the current study (9.34 ± 5.16 mL).

A comparison of the total SI fluid volume shows that the volumes measured in the current study are larger than previously reported volumes in children by Papadatou-Soulou et al. [14]. Similar to the results for the gastric fluid volume, this discrepancy might be attributed to differences in the technique used for fluid integration.

When comparing the adolescent population assessed in the present study to data available for the adult population, the average adolescent SI fluid volume of 94.01 ± 70.39 mL is comparable to published data by Schiller et al. [32] (Figure 5). However, a comparison with other studies shows a higher average volume present for the adolescent population compared to the adult population. This discrepancy may originate from differences in fasting time. All studies in the adult population described in the literature concern a prospective study with highly controlled conditions, including fasting time. For the current retrospective study using historical clinical practice data, control over adherence to the hospital MRI protocol is very limited. Consequently, shorter fasting times with incomplete GI transit and water reabsorption are possible. The impact of such outliers is possibly reflected by the average SI fluid volume for adolescents (94.01 mL) being much higher than the median SI fluid volume (62.9 mL), indicating a skewedness of the data. The median adolescent SI fluid volume is more in line with the previously reported average fluid volumes of the adult population.

#### 3.4.3. Colon

The average fluid volumes measured in the colon of children are clearly lower than in a previous MRI study by Goelen et al. [13]. As addressed by Goelen et al. [13], the average colonic volume in their study was highly affected by outliers. When comparing median colonic fluid volumes, the data by Goelen et al. [13] (0.80 mL) are more in line with the median volume for all children in the present study (0.49 mL). A comparison for the individual subpopulations was not possible as only an average for the whole pediatric population was reported by Goelen et al.

As no specific age-related pattern was found for the colonic fluid volumes in pediatrics, a direct comparison with the adult population is made. When comparing average fluid volumes, pediatric colonic fluid volumes seem to be in line with previously reported volumes in adults, as few to no fluid pockets are typically found in studies using a similar technique [32,33,34] (Figure 5).

### 3.5. Weight-Corrected Gastric Fluid Volumes in the Literature

Although data on absolute pediatric gastric fluid volumes are scarce in the literature, multiple papers reported weight-corrected gastric fluid volumes (WCGFV) in children generated in the context of anesthesia, where gastric fluid is often aspirated or measured using ultrasound or sometimes using MRI [25]. These WCGFVs have been thoroughly reviewed by Andersson et al. [45] and are summarized in Figure 5. In general, the WCGFV generated in the present study is in line with available literature data.

Andersson et al. [45] showed the dependency of the gastric fluid volume corrected for body weight on the fasting time. As can be seen from Figure 5, the data from the current study are relatively higher for the toddler, preschool children, and school-age children pediatric subpopulations. These higher data could indicate that the included patients drank some fluid until recently (1–3 h) before imaging, as children aged 1–11 years become more irritated when fasted [25]. Consequently, it has to be expected that in clinical practice, younger children (aged 1–11 years) were allowed to drink until relatively close before the examination. This is in contrast to the strict 8 h fasting time that is often used in clinical studies for adults.

Additionally, some observed differences might originate from the technique used. When using an aspiration technique where gastric fluid is extracted from the body, it is possible that some fluid remains in the stomach. MRI considers all free fluid present though its accuracy is, as previously mentioned, dependent on the technique used. As no fluid integration protocol was available for the MRI studies, their technique could not be compared to the one used in this study.

### 3.6. GI Fluid Volumes in Simulation Tools for Drug Absorption

The importance of GI fluid volumes is obvious during in vitro dissolution testing. As assessed by Kostewicz et al. [46], the current quality control dissolution setups do not use a fluid volume representative of the in vivo situation. Using representative volumes is especially relevant for newly developed drugs that tend to suffer from solubility problems, often making the available volume of fluid a limitation. Consequently, knowledge of the composition and available volume of (GI) fluid for dissolution is crucial to adequately predict drug absorption. As such, the use of a physiologically relevant volume for dissolution should also be extended to pediatric (biorelevant) in vitro models. The data generated in this study could allow better and more representative scaling. Using more relevant in vitro models, new (pediatric) formulations and drugs can be screened faster and in a more physiologically relevant manner. Additionally, the data on GI fluid volumes could also help in the development of a pediatric BCS classification system.

Additionally, a major use of the data presented in this publication is as input for PBPK modeling of drug absorption. Gastroplus and Simcyp, currently the most used PBPK modeling platforms, implement different approaches to calculate GI fluid volumes. In Simcyp, the resting gastric fluid volume is calculated based on the gastric fluid secretion and gastric emptying rate. These parameters are age dependent but fixed by the software based on Simcyp in-house data and literature research [47]. In Gastroplus, fluid volumes are calculated based on age-dependent dimensions of the GI tract and the assumption that a fixed age-independent percentage of the intestinal tract is filled with fluid [28]. This dataset could help to better describe the physiological state of the fasted GI tract in children and improve the relevance of the physiological models.

**Figure 6:**
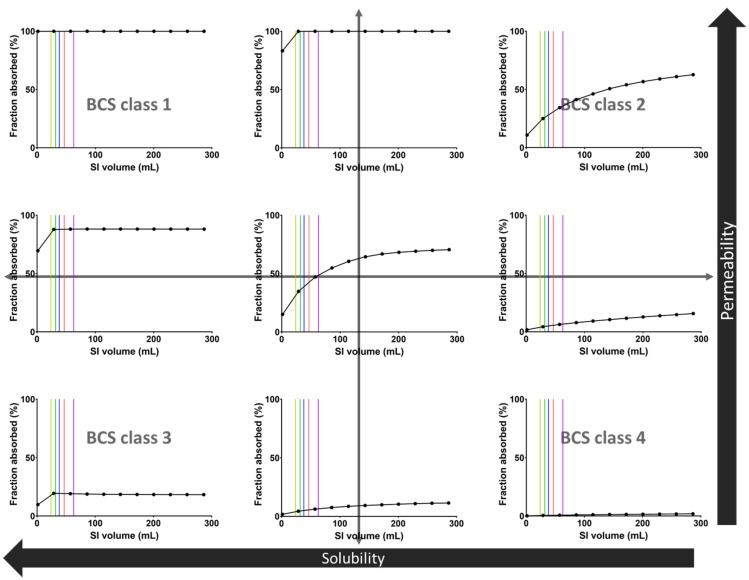
Sensitivity analysis of the fraction absorbed of 9 hypothetical drugs as a function of the fluid volume in the SI. Vertical lines indicate the median SI fluid volumes for the different pediatric subpopulations: yellow = infant, green = toddler, blue = preschool child, red = school-age child, purple = adolescent.

In the framework of the present study, PBPK modeling was used to explore the potential impact of physiologically relevant GI fluid volumes on drug absorption. To this end, models for hypothetical drugs representing the different BCS classes and their boundaries were developed (Figure A1). Next, the sensitivity of these drugs’ fraction absorbed to the volume available in the SI was evaluated and is depicted in Figure 6.

The sensitivity of the fraction absorbed to the available SI fluid volume strongly depends on the BCS class of the hypothetical drugs (Figure 6). The median pediatric fluid volumes measured in the present study are within the sensitive area for medium to low solubility compounds, indicating that physiologically relevant variations in fluid volume may affect the extent of drug absorption. As expected, low solubility drugs (BCS2 and BCS 4) are more sensitive to GI fluid and have a fraction absorbed dependent on the fluid volume. In contrast, high-solubility drugs (BCS 1 and BCS 3) seem to have a fraction absorbed largely independent of the fluid volume. Lastly, medium solubility drugs (boundary) seem to have a fraction absorbed dependent on the fluid volume unless the permeability is high.

These sensitivity simulations indicate that the use of biorelevant GI fluid volumes is important when evaluating poorly soluble drugs since it may substantially affect their absorption. In clinical practice, such simulations should obviously take into account other factors influencing drug exposure for poorly soluble drugs, including the use of advanced formulations to reduce solubility issues and possible dose reductions in younger children. 

## 4. Conclusions

This retrospective study successfully quantified GI fluid volumes in the pediatric population resulting in the largest and most detailed dataset currently available. We analyzed pediatric abdominal MRI images gathered by the University Hospitals Leuven for GI fluid volumes using a method that has already been successfully used in adults. In addition, the existing method was used to differentiate between fluid pockets in the duodenum, jejunum, and ileum instead of considering these parts of the SI as a whole.

In general, the median fluid volumes increased with age in all GI segments except for the ileum and colon. Additionally, an increase in fluid volumes was observed in more distal SI segments except for the adolescent subpopulation, where the median fluid volume was lower in the ileum than in the jejunum. Most of the colonic fluid pockets were located in the first part of the colon (ascending colon).

PBPK simulations using hypothetical compounds suggested that the range of pediatric fluid volumes observed in this study can have a substantial impact on the absorption of poorly soluble drugs. This highlights the importance of using physiologically representative fluid volumes in in vitro and in silico simulation tools to predict drug absorption in children.

Lastly, the observed GI fluid volumes appeared to correlate only poorly with easy-to-measure body characteristics (BMI, weight, height, SI length). This observation suggests the need for caution when scaling GI fluid volumes using these clinical estimators.

## Figures and Tables

**Table 1:** Exclusion criteria with their respective number of excluded patients.

Exclusion Criteria	Number of Patients
Start	579
Fluid-fed state	19
Non-fasted state	171
Image quality	158
Pathology related	
Ascites	3
Cholangitis	1
Cholecystectomy	31
Feeding tube	4
Haemochromatosis	1
Inflammatory bowel disease	8
Large tumor	22
Pancreatitis	14
Stoma	3
Extensive hepatosplenomegaly	4
Final dataset	140

**Table 2:** Demographics of the included patients.

Subpopulation	Included Children	Average BMI (kg/m^2^) (SD)	Sex Distribution	
			F	M
Infant (0.1–1 year)	5	16.20 (0.69)	3	2
Toddler (1–2 year)	15	16.31 (1.23)	9	6
Preschool child (2–5 year)	82	15.41 (1.59)	37	45
School-age child (6–11 year)	23	17.84 (3.53)	13	10
Adolescent (12–16 year)	15	20.10 (3.00)	11	4

## Data Availability

Not applicable.

## Appendix A

**Table A1:** Input parameters of the PBPK model.

Parameter	Input Value
Molecular weight	300
LogP (neutral)	1
Dose (mg)	100
Dosage form	Immediate release: tablet
Dose volume (mL)	250
pH for ref. solubility	7
Solubility (mg/mL)	See Figure A1
Mean precipitation time (sec)	900
Diff. coeff. (10^−5^ × cm^2^/s)	0.75
Drug particle density (g/mL)	1.2
Peff (10^−4^ × cm/s)	See Figure A1
pKa	N/A
Simulation length (h)	24.00
