# Correction: Van der Veken et al. Gastrointestinal Fluid Volumes in Pediatrics: A Retrospective MRI Study. *Pharmaceutics* 2022, *14*, 1935

**DOI:** 10.3390/pharmaceutics15092323

**Published:** 2023-09-15

**Authors:** Matthias Van der Veken, Michael Aertsen, Joachim Brouwers, Cordula Stillhart, Neil Parrott, Patrick Augustijns

**Affiliations:** 1 Drug Delivery and Disposition, KU Leuven, Gasthuisberg O&N II, Herestraat 49—Box 921, 3000 Leuven, Belgium; 2 Department of Imaging and Pathology, Clinical Department of Radiology, University Hospitals KU Leuven, 3000 Leuven, Belgium; 3 Formulation & Process Sciences, F. Hoffmann-La Roche Ltd., 4070 Basel, Switzerland; 4 Pharmaceutical Sciences, Roche Pharma Research and Early Development, Roche Innovation Centre Basel, 4070 Basel, Switzerland

## Error in Figure

In the original publication [1], there was a mistake in Figure 5 as published. The corrected Figure 5 caption appears below. The original publication has also been updated.

Previous version: 
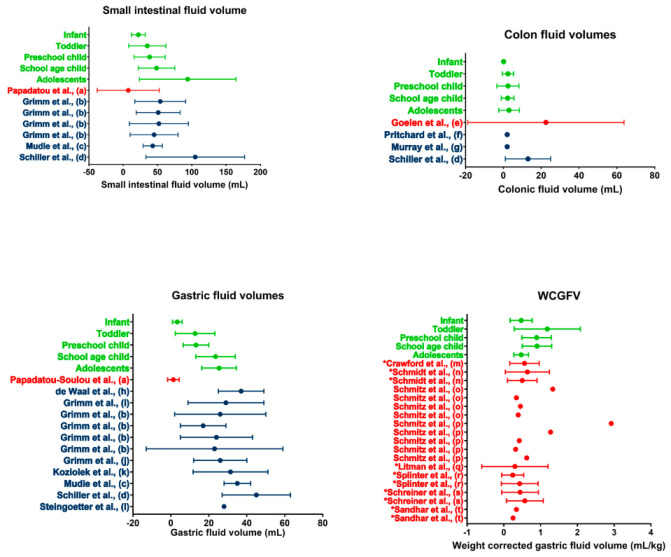
 **Figure 5.** Literature comparison for SI, colon, gastric, and WCGV. Dots indicate average volumes; error bars indicate standard deviations. Data obtained in the present study (green) are compared to data from other studies on children (red) or adults (blue). WCGV measured from fluid aspirations are indicated by a * in front of the reference; other WCGVs are measured using MRI. (a [14], b [20], c [17], d [31], e [13], f [32], g [33], h [22], i [21], j [35], k [26], l [36], m [37], n [38], o [39], p [40], q [41], r [42], s [43], t [44]).

Updated version: 
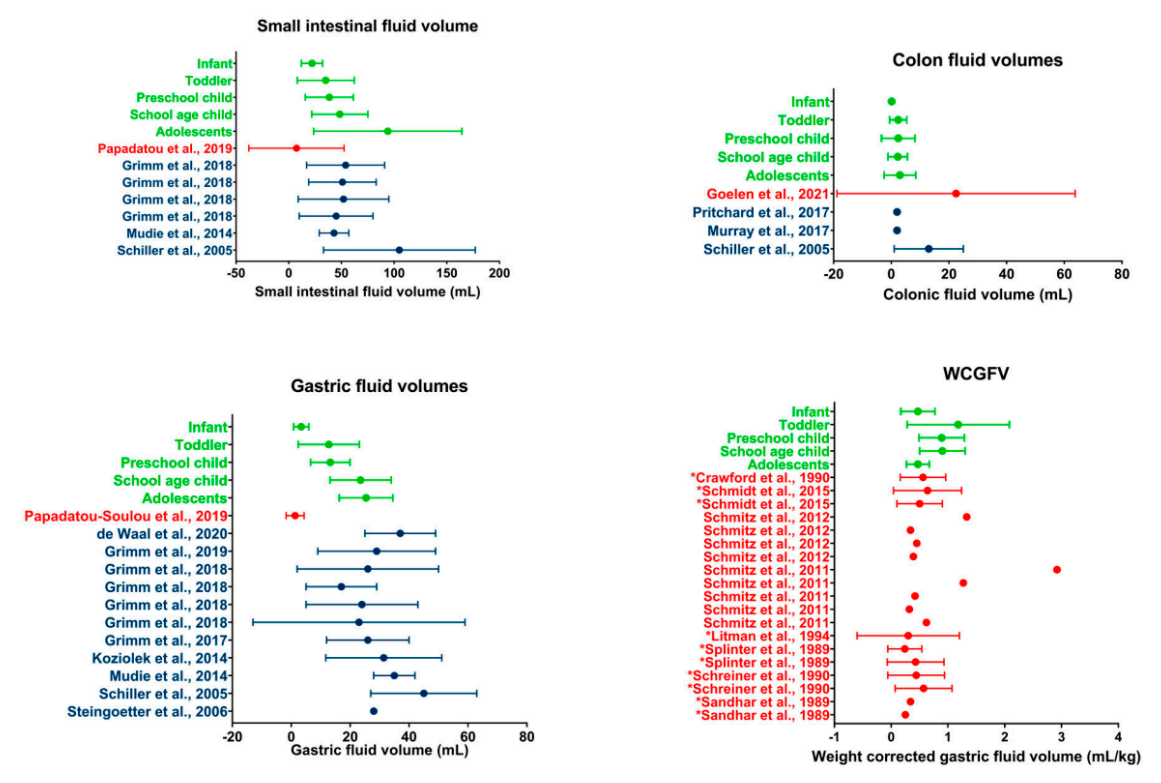
 **Figure 5.** Literature comparison for SI, colon, gastric, and WCGV. Dots indicate average volumes; error bars indicate standard deviations. Data obtained in the present study (green) are compared to data from other studies on children (red) or adults (blue). WCGV measured from fluid aspirations are indicated by a * in front of the reference; other WCGVs are measured using MRI [13,14,17,20–22,26,32–44].
